# Testican-2: from glomerular activity to circulating biomarker

**DOI:** 10.3389/fphys.2026.1953293

**Published:** 2026-10-26

**Authors:** Jiao Luo, Heng Sun, Li-Yan Jiang, Xin-Hua Xiao

**Affiliations:** 1 Department of Metabolism and Endocrinology, The First Affiliated Hospital, Hengyang Medical School, University of South China, Hengyang, China; 2 Clinical Research Center for Obesity in Hunan Province, Hengyang, China

**Keywords:** glomerular basement membrane, glomerular disease, integrin αvβ3, kidney biomarker, podocyte, *SPOCK2*, Testican-2, vitronectin

## Abstract

Testican-2, encoded by *SPOCK2*, is a secreted extracellular matrix-associated proteoglycan involved in podocyte and glomerular biology. The kidney contributes to the circulating Testican-2 pool, with podocytes representing a major renal source. Higher circulating levels are generally associated with better-preserved kidney function, less proteinuria, more favorable renal outcomes, and milder glomerular pathology, although these relationships vary across disease settings. *In vitro* and rodent studies show that Testican-2 binds vitronectin, restrains excessive integrin αVβ3 activation, and attenuates podocyte and filtration barrier injury. However, plasma abundance cannot yet be assumed to reflect the biologically active glomerular pool, because circulating levels may also be influenced by extracellular retention, glycosylation, proteolytic processing, and clearance. This uncertainty limits both biomarker interpretation and therapeutic translation. This Mini Review integrates clinical and experimental evidence to clarify what circulating Testican-2 may represent and identify the studies needed to connect plasma measurements with local glomerular activity.

## 1 Introduction

The integrity of the glomerular filtration barrier depends on coordinated interactions between podocytes and the glomerular basement membrane (GBM) (Suh and Miner, 2013). The podocyte–GBM interface is a specialized extracellular compartment where cell adhesion, matrix organization, and receptor signaling converge to maintain glomerular homeostasis. Disruption of this interface leads to foot process effacement, podocyte detachment, proteinuria, and progressive loss of kidney function (Wharram et al., 2005; Tian et al., 2014; Nagata, 2016). Extracellular proteins that regulate ligand presentation or receptor engagement at this site may therefore influence both glomerular homeostasis and disease progression.

Testican-2, encoded by *SPOCK2*, has emerged as a candidate regulator of this interface through converging clinical and experimental evidence. Paired renal arterial and venous proteomic studies demonstrated net release of Testican-2 from the kidney (Ngo et al., 2020). Single-nucleus RNA sequencing identified podocytes as the predominant renal cell type expressing *SPOCK2*, while histological studies localized Testican-2 along the glomerular capillary wall near the podocyte-facing aspect of the GBM (Ngo et al., 2020). Higher circulating levels have generally been associated with better-preserved kidney function, more favorable renal outcomes, and less glomerulosclerosis (Ngo et al., 2020; Wen et al., 2023; Kim et al., 2024), although membranous nephropathy shows a distinct disease-specific pattern (Kim et al., 2026). Experimental studies further showed that podocyte-derived Testican-2 binds vitronectin and limits excessive integrin αVβ3 activation (Wen et al., 2026).

These observations concern related but non-equivalent aspects of Testican-2 biology. Matrix-associated Testican-2 may act locally at the podocyte–GBM interface, whereas plasma levels may reflect the combined effects of renal and extrarenal production, extracellular retention, release, molecular processing, and clearance. Mendelian randomization supports an effect of kidney function on circulating Testican-2 (Matías-García et al., 2021). Whereas a recent review surveyed the broader multi-organ biology of Testican-2, this Mini Review focuses on the kidney-specific relationship between local Testican-2 activity at the podocyte–GBM interface and its circulating biomarker implications. We integrate clinical and experimental evidence to clarify what circulating Testican-2 may represent and identify the studies required to link plasma abundance with local glomerular activity (Wang et al., 2026).

## 2 Renal source and distribution of Testican-2

The molecular forms of Testican-2 present in the kidney remain incompletely characterized. Testican-2 can undergo N-linked glycosylation and cell-dependent glycosaminoglycan modification (Schnepp et al., 2005). Proteolytic processing and matrix association may further influence its local retention and release, but kidney-specific cleavage products have not been systematically characterized (Wang et al., 2026).

Most kidney cohort studies measured relative Testican-2 abundance using aptamer-based platforms (Ngo et al., 2020; Wen et al., 2023; Chen et al., 2024; Kim et al., 2024, 2026). Whether these measurements are equivalent to antibody-based or mass-spectrometry measurements remains unknown. More broadly, affinity-based protein measurements can show substantial inter-platform discordance and may be affected by epitope accessibility (Nicholas et al., 2026).

Renal arteriovenous sampling first showed an increase in circulating Testican-2 from the arterial to the renal venous compartment, demonstrating net release from the kidney. This gradient does not quantify the renal contribution to the total plasma pool or identify the route by which the protein reaches renal venous blood (Ngo et al., 2020).

Single-nucleus RNA sequencing of human kidney tissue identified podocytes as the predominant renal cell type expressing *SPOCK2*, while microscopy localized Testican-2 along glomerular capillary loops near the podocyte-facing aspect of the GBM. Podocyte-specific *Spock2* deletion subsequently reduced circulating Testican-2, providing direct evidence that podocytes contribute to the systemic pool. They can therefore be considered a major renal source, although their fractional contribution and possible context-dependent expression by other renal cells remain undefined (Ngo et al., 2020; Wen et al., 2026).

Localization near the GBM is consistent with a matricellular regulatory role rather than that of a major structural component. At this interface, Testican-2 can influence extracellular ligand availability and receptor engagement, as demonstrated for vitronectin. How much locally retained protein subsequently enters plasma remains unknown. Its renal molecular forms and release kinetics remain poorly characterized, and it is unclear whether circulating levels reflect Testican-2 abundance or activity in the glomerulus (Wang et al., 2026; Wen et al., 2026). These relationships are summarized in **Figure 1**.

**Figure 1:**
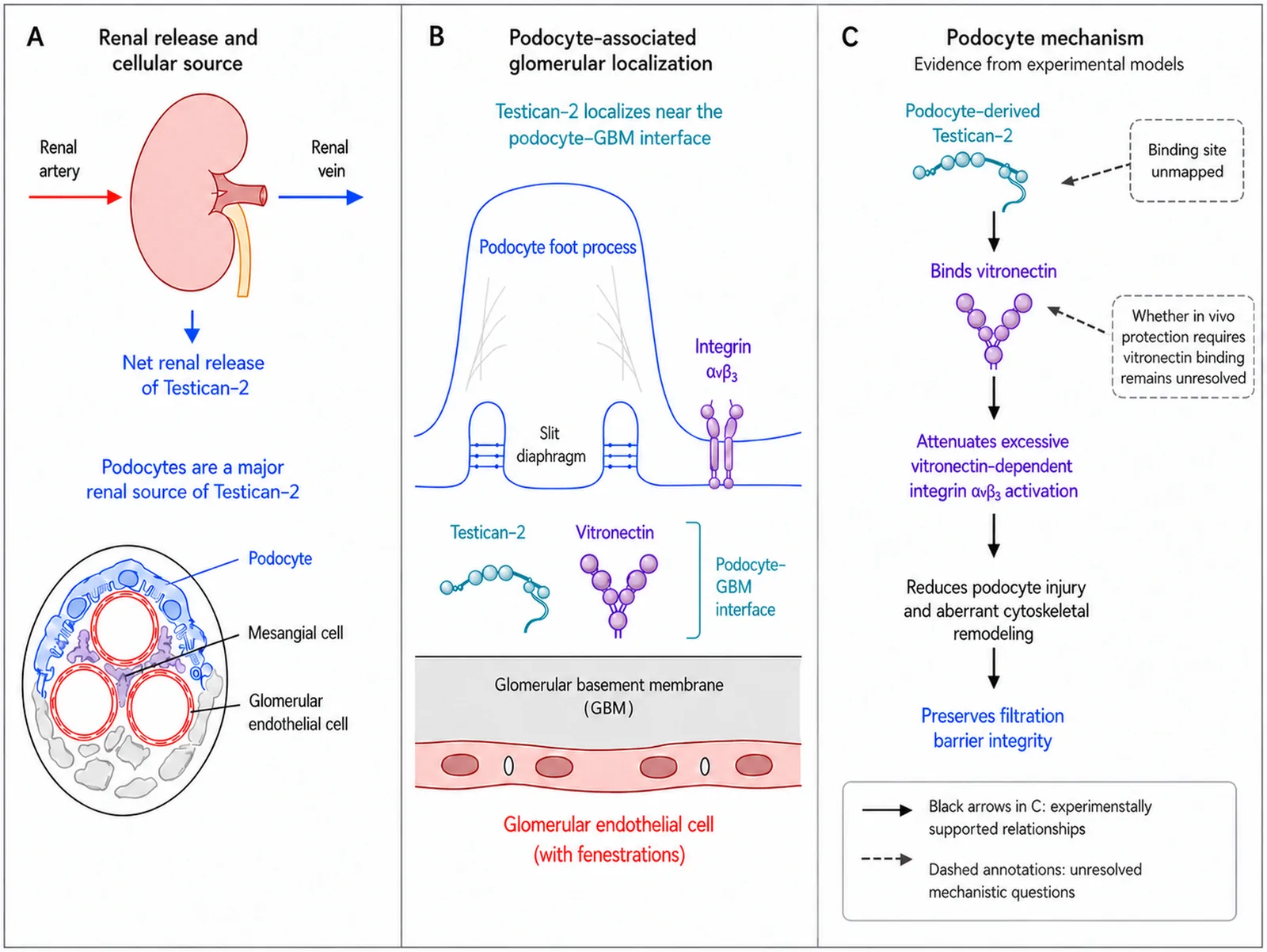
Renal source, glomerular localization, and podocyte actions of Testican-2. **(A)** The kidney shows net release of Testican-2, with podocytes representing an important renal source. Podocytes, glomerular endothelial cells, and mesangial cells are indicated. **(B)** Testican-2 localizes near the podocyte–glomerular basement membrane (GBM) interface. **(C)** Testican-2 binds vitronectin and attenuates vitronectin-dependent integrin αvβ3 activation, thereby limiting podocyte injury and aberrant cytoskeletal remodeling and preserving filtration-barrier integrity. Solid arrows indicate experimentally supported relationships, whereas dashed annotations indicate unresolved mechanistic questions, including the Testican-2 binding site and whether *in vivo* protection requires vitronectin binding. GBM, glomerular basement membrane.

## 3 Protective actions of Testican-2 in the glomerulus

Vitronectin provides the best-supported mechanistic link between Testican-2 and the podocyte–GBM interface. It binds integrin αVβ3, whose excessive activation in podocytes promotes motility, actin remodeling, loss of adhesion, foot process injury, and proteinuria (Wei et al., 2008). Immunoprecipitation followed by mass spectrometry identified vitronectin as a Testican-2-interacting extracellular protein. Direct binding was subsequently confirmed by biolayer interferometry, and competition assays showed that Testican-2 reduced vitronectin engagement with integrin αVβ3. In cultured podocytes, this was accompanied by lower integrin activation, better preservation of actin organization and adhesion, and less adriamycin-induced injury (Wen et al., 2026).

How Testican-2 modifies vitronectin remains unresolved. Its binding region has not been mapped, and current evidence does not distinguish whether it restricts access to the arginine–glycine–aspartate (RGD) motif, changes vitronectin conformation, or alters its presentation within the extracellular matrix. Deglycosylation weakens but does not abolish the interaction, suggesting that carbohydrate chains facilitate rather than solely determine binding. Downstream effects on focal adhesion signaling, Src-family kinases, and Rho GTPases have not been directly examined (Wen et al., 2026).

The protective phenotype was reproduced *in vivo*. Both global and podocyte-specific *Spock2* deletion increased susceptibility to adriamycin-induced glomerular injury, with greater albuminuria and foot process effacement; global deletion also exacerbated streptozotocin-induced injury (Wen et al., 2026). Podocyte-specific deletion establishes podocytes as an important source of protective Testican-2, but it does not restrict the protein’s actions to the producing cell. Once secreted, Testican-2 may influence neighboring podocytes, other glomerular cells, or the organization of the GBM (Wen et al., 2026).

In *Spock2*-deficient mice, adenoviral expression of *Spock2* increased circulating Testican-2. The restored protein reached the glomerulus, bound vitronectin, reduced the vitronectin–integrin αVβ3 interaction, and attenuated podocyte and filtration barrier injury (Wen et al., 2026). These findings implicate the vitronectin–integrin αVβ3 pathway in the protective effects of Testican-2, but whether vitronectin binding is required for protection *in vivo* remains unclear. The mechanistic and rescue evidence comes largely from a single study and requires independent replication.

A Testican-2 variant that retains secretion, extracellular stability, and glomerular localization but lacks vitronectin-binding activity would provide a more direct test of the proposed mechanism. Failure of such a variant to rescue injury would support a requirement for vitronectin binding. Preserved protection would instead point to additional ligands, regulation of extracellular proteolysis, or matrix-organizing functions. Selective restoration in podocytes and delivery restricted to the circulation could further distinguish the cellular source of Testican-2 from its site of action.

Podocytes are not the only glomerular cells that respond to Testican-2. In primary human glomerular endothelial cells, Testican-2 increased tube formation and migration without increasing proliferation. These effects were accompanied by increased matrix metalloproteinase-2/-9 activity in the culture medium (Ngo et al., 2020), suggesting that extracellular matrix proteolysis may contribute to migration, although this has not been established. Testican-2 directly affects glomerular endothelial cells *in vitro*, but its role in the glomerular endothelium *in vivo* remains unclear. Whether the vitronectin–integrin αVβ3 pathway mediates the endothelial effects of Testican-2 requires further investigation.

## 4 Clinical evidence for circulating Testican-2

Evidence that the kidney releases Testican-2 into the circulation led to its evaluation in kidney disease (Ngo et al., 2020). Clinical studies have therefore examined whether circulating Testican-2 tracks kidney function, predicts progression, and reflects glomerular injury across different disease settings (**Table 1**).

**Table 1:** Integrated evidence for renal Testican-2.

Evidence domain	Evidence type	Sample/tissue	Key evidence	Interpretation	Main gap
Renal origin and localization	Human proteomic analysis, renal expression/localization studies, and podocyte-specific knockout in mice	Paired human renal arterial and venous plasma; human kidney tissue; mouse serum and kidney tissue	Net renal release; podocyte-enriched *SPOCK2* expression; localization near the podocyte-facing GBM; reduction in circulating Testican-2 after podocyte-specific *Spock2* deletion (Ngo et al., 2020; Wen et al., 2026)	Supports a kidney-derived, podocyte-associated Testican-2 pool	The fractional podocyte contribution to the total circulating pool is unknown
Circulating associations	Human observational proteomic and longitudinal cohort studies	Human plasma; human serum in AASK	Higher Testican-2 was associated with higher eGFR, lower albuminuria, and lower kidney-failure risk (Ngo et al., 2020; Matías-García et al., 2021; Wen et al., 2023; Chen et al., 2024)	Candidate marker of preserved renal status	Incremental value beyond eGFR and albuminuria is unproven
Glomerular pathology and disease heterogeneity	Human plasma proteomics, kidney biopsy/glomerular expression analysis, and diagnostic modeling	Human plasma; kidney biopsy and glomerular tissue	Higher circulating Testican-2 was associated with less glomerulosclerosis. In membranous nephropathy, plasma Testican-2 and glomerular *SPOCK2* expression were increased, and Testican-2 combined with MGT5A improved diagnostic discrimination (Kim et al., 2024, 2026)	Testican-2 may carry different biological and diagnostic meanings across glomerular diseases	Histopathologic evidence is largely cross-sectional, and diagnostic performance in membranous nephropathy requires external validation and comparison with established serological markers
Local protective mechanism	*In vitro* protein-binding and podocyte functional assays	Purified proteins; cultured human podocytes	Testican-2 bound vitronectin and reduced vitronectin-dependent integrin αVβ3 activation and podocyte injury (Wen et al., 2026)	Best-supported mechanism of local glomerular protection	Binding interface and downstream signaling remain unresolved
Genetic and rescue evidence	Global and podocyte-specific knockout and adenoviral rescue in mice	Mouse serum, urine, and kidney tissue	Global or podocyte-specific *Spock2* loss aggravated injury; restoration produced renal target engagement and partial rescue (Wen et al., 2026)	Supports an endogenous and partly reversible protective function	Local versus circulating contribution, active form, dose, and safety remain undefined

Clinical proteomic studies measured relative aptamer-based abundance. AASK used human serum; the other listed clinical cohorts used plasma. Mechanistic and rescue evidence comes mainly from one study and requires independent replication. AASK, African American Study of Kidney Disease and Hypertension; eGFR, estimated glomerular filtration rate; GBM, glomerular basement membrane; MGT5A, alpha-1,6-mannosylglycoprotein 6-beta-N-acetylglucosaminyltransferase.

### 4.1 Kidney function and outcomes

In community-based cohorts, higher circulating Testican-2 was associated with higher estimated glomerular filtration rate (eGFR), slower subsequent loss of kidney function, and a lower risk of incident chronic kidney disease (Ngo et al., 2020). A transethnic proteomic analysis reproduced the positive relationship with eGFR and examined its direction using Mendelian randomization (MR) (Matías-García et al., 2021). The MR results supported an effect of eGFR on circulating Testican-2 but provided no statistically significant evidence for the reverse direction (Matías-García et al., 2021). Circulating abundance is therefore more appropriately interpreted as a correlate of preserved kidney status than as evidence that the plasma protein directly protects the kidney. This interpretation remains compatible with a local protective role for matrix-associated Testican-2 at the podocyte–GBM interface (Wen et al., 2026).

Across African American Study of Kidney Disease and Hypertension (AASK), Chronic Renal Insufficiency Cohort (CRIC), and Atherosclerosis Risk in Communities (ARIC), higher circulating Testican-2 was generally associated with less proteinuria or albuminuria and a lower risk of subsequent kidney failure (Wen et al., 2023). After adjustment including proteinuria, the association with kidney failure remained significant in AASK and ARIC but not in CRIC, although the pooled association remained significant (Wen et al., 2023). A separate proteome-wide analysis found that Testican-2 was the only protein significantly associated with a lower risk of incident kidney failure in the pooled analysis (Chen et al., 2024).

AASK was included in both the incident kidney failure and proteome-wide kidney failure analyses, while the Boston Kidney Biopsy Cohort contributed to several histopathologic and disease-classification studies (Wen et al., 2023; Chen et al., 2024; Kim et al., 2024, 2026). Differences in affinity platform and probe recognition may further contribute to variation between studies (Nicholas et al., 2026). These limitations should be considered before a common clinical threshold is proposed.

Adding Testican-2 to fully adjusted models or the Kidney Failure Risk Equation did not improve discrimination or risk reclassification for incident end-stage kidney disease (ESKD) (Wen et al., 2023). eGFR reflects filtration capacity, whereas albuminuria indicates filtration barrier dysfunction but is influenced by several renal and systemic processes (Levey et al., 2015). Circulating Testican-2 could contain additional information related to podocyte or extracellular matrix integrity, yet adjusted associations alone do not demonstrate improved discrimination, calibration, risk classification, or clinical decision-making (Tangri, 2011; Tangri et al., 2016). Clinically usable cutoffs, within-person variability, sample stability, and treatment-related changes also remain undefined (Wen et al., 2023).

### 4.2 Glomerular pathology and disease context

Biopsy data provide a closer link between circulating Testican-2 and glomerular structure. In the Boston Kidney Biopsy Cohort, higher plasma levels were associated with less glomerulosclerosis after adjustment for eGFR, proteinuria, and demographic factors (Kim et al., 2024). The analysis was cross-sectional, however, and cannot determine whether reduced circulating Testican-2 precedes podocyte loss or reflects established chronic damage.

Tissue studies suggest that this relationship differs among glomerular diseases. Glomerular Testican-2 immunofluorescence intensity was significantly lower in diabetic kidney disease, showed a nonsignificant decrease in focal segmental glomerulosclerosis, and did not differ from controls in tubulointerstitial nephropathy (Wen et al., 2026). Membranous nephropathy showed a different pattern. Plasma Testican-2 and glomerular *SPOCK2* expression were increased, and the combination of Testican-2 and alpha-1,6-mannosylglycoprotein 6-beta-N-acetylglucosaminyltransferase (MGT5A) improved discrimination of membranous nephropathy from other biopsy diagnoses in the Boston Kidney Biopsy Cohort and Nephrotic Syndrome Study Network (NEPTUNE) (Kim et al., 2026).

Anti-phospholipase A2 receptor (anti-PLA2R) testing supports the diagnosis of PLA2R-associated membranous nephropathy (MN), but a negative serum result does not exclude MN (Debiec and Ronco, 2011; Bobart et al., 2021; Rovin et al., 2021; Zee et al., 2025). Anti-PLA2R testing was not included in the models assessing Testican-2 and MGT5A. The combination’s added diagnostic value beyond anti-PLA2R testing remains unclear, and its performance in anti-PLA2R-negative patients was not evaluated separately (Kim et al., 2026). Further studies should therefore include anti-PLA2R testing to assess the combination’s added diagnostic value, with separate analyses in anti-PLA2R-negative patients.

More broadly, these disease-specific findings suggest that circulating Testican-2 should be interpreted in the context of the underlying glomerular disease. Longitudinal studies with paired plasma and tissue measurements are needed to determine whether Testican-2 changes with disease activity, treatment response, chronic podocyte loss, or remodeling of the GBM.

## 5 Local and circulating Testican-2

Current evidence allows three overlapping interpretations of plasma Testican-2. Under a coupled-source model, podocytes contribute to both the matrix-associated glomerular pool and the protein entering renal venous blood. The renal arteriovenous gradient, podocyte-enriched *SPOCK2* expression, lower circulating levels after podocyte-specific *Spock2* deletion, and associations with glomerular pathology are consistent with a shared source (Ngo et al., 2020; Kim et al., 2024; Wen et al., 2026). The fraction of plasma Testican-2 derived from podocytes has not been quantified, however, and whether circulating levels reflect local protective activity remains unclear.

A second interpretation places kidney status upstream of the circulating signal (Matías-García et al., 2021). Under this model, higher levels could reflect preserved kidney function and be associated with favorable outcomes without implying that circulating Testican-2 itself mediates protection. This interpretation remains compatible with a local role for matrix-retained Testican-2 at the podocyte–GBM interface.

Partial uncoupling is also plausible. Matrix retention and glycosylation may influence local availability and vitronectin binding, whereas proteolytic processing, urinary loss, extrarenal production, systemic clearance, and epitope accessibility may alter what reaches plasma or is detected by current assays (Schnepp et al., 2005; Nicholas et al., 2026; Wang et al., 2026). The higher plasma Testican-2 levels and glomerular *SPOCK2* expression reported in membranous nephropathy highlight a disease-specific pattern. Whether increased glomerular expression or GBM remodeling contributes to the higher circulating levels remains unclear (Kim et al., 2026).

These interpretations lead to different experimental expectations. A coupled source should produce parallel changes in podocyte abundance, renal *SPOCK2* expression, glomerular protein, and plasma Testican-2 after kidney function is taken into account. A renal-status signal should track eGFR or broader tissue integrity more closely than local vitronectin engagement. Uncoupling would appear as discordant tissue and plasma measurements, particularly when matrix remodeling or protein processing changes.

Paired longitudinal tissue and plasma samples, quantitative source tracing, and assays that resolve molecular forms are needed to distinguish these possibilities. Source-selective labeling or restoration in podocytes, combined with arterial, renal venous, urinary, and glomerular measurements, would be especially informative. Until such evidence is available, circulating Testican-2 is best regarded as a candidate marker of preserved renal status rather than a direct readout of local glomerular activity.

## 6 Discussion

The kidney releases Testican-2 into the circulation, and current evidence identifies podocytes as a major renal source (Ngo et al., 2020; Wen et al., 2026). Higher circulating levels generally accompany preserved kidney function and less glomerulosclerosis (Wen et al., 2023; Kim et al., 2024). Membranous nephropathy shows a different pattern (Kim et al., 2026). Experimental studies further implicate the vitronectin–integrin αVβ3 pathway in the protective effects of Testican-2 (Wen et al., 2026).

These observations should not be treated as interchangeable. Clinical associations do not demonstrate that circulating Testican-2 directly protects the kidney, and a local extracellular mechanism does not establish that plasma abundance reflects the active protein at the podocyte–GBM interface.

The apparent breadth of the evidence also exceeds its degree of independence. Several publications reused AASK, the Boston Kidney Biopsy Cohort, or the original renal arteriovenous cohort (Ngo et al., 2020; Wen et al., 2023; Chen et al., 2024; Kim et al., 2024). Most mechanistic evidence comes from one integrated study combining binding assays, cultured podocytes, genetic models, and restoration experiments (Wen et al., 2026). Agreement across these approaches is encouraging, but independent replication across cohorts, laboratories, assay platforms, and experimental systems remains limited.

Clinical translation will first require a clearer definition of what current assays measure. Aptamer-based platforms, immunoassays, and mass spectrometry may differ in their recognition of intact, glycosaminoglycan-bearing, or processed forms of Testican-2. Cross-platform analysis of the same samples and molecularly defined reference preparations are therefore needed before absolute thresholds can be compared (Kirsher et al., 2025; Nicholas et al., 2026). Subsequent studies should test whether Testican-2 improves prediction beyond eGFR, albuminuria, and validated clinical models. The intended use must also be specified. A marker for long-term kidney failure risk and a diagnostic marker for membranous nephropathy require different study populations, comparators, and performance criteria (Pepe et al., 2008). The diagnostic performance of Testican-2 combined with MGT5A requires further independent validation, including assessment of its added value beyond anti-PLA2R testing in antibody-positive and antibody-negative patients (Rovin et al., 2021; Kim et al., 2026).

Therapeutic development remains at the proof-of-concept stage. Published restoration experiments increased Testican-2 through adenoviral *Spock2* expression rather than a clinically deployable protein or targeted formulation (Wen et al., 2026). A Testican-2 preparation would need a defined active form, adequate exposure at the podocyte-facing GBM, measurable engagement of vitronectin, and an acceptable safety profile. Raising the plasma concentration alone may not reproduce a matrix-dependent local effect. Nonrenal distribution, immunogenicity, durability, and interference with physiological vitronectin–integrin signaling will also require evaluation.

The most informative next studies would combine longitudinal plasma and tissue sampling with quantitative tracing of podocyte-derived Testican-2 and molecularly resolved proteomic or glycoproteomic analysis. Rescue using a Testican-2 variant that retains secretion and glomerular localization but lacks vitronectin-binding activity would test whether this interaction is required *in vivo*. These studies would help clarify the potential of Testican-2 as a context-specific biomarker, a locally acting extracellular therapeutic candidate, or both.
